# Bleeding disorders in the tribe: result of consanguineous in breeding

**DOI:** 10.1186/1750-1172-5-23

**Published:** 2010-09-07

**Authors:** Munira Borhany, Zaen Pahore, Zeeshan ul Qadr, Muhammad Rehan, Arshi Naz, Asif Khan, Saqib Ansari, Tasneem Farzana, Muhammad Nadeem, Syed Amir Raza, Tahir Shamsi

**Affiliations:** 1 Haemostasis & Thrombosis department of National Institute of Blood Disease & Bone Marrow Transplantation, Karachi, Pakistan

## Abstract

**Objective:**

To determine the frequency and clinical features of bleeding disorders in the tribe as a result of consanguineous marriages.

**Design:**

Cross Sectional Study

**Introduction:**

Countries in which consanguinity is a normal practice, these rare autosomal recessive disorders run in close families and tribes. Here we describe a family, living in village Ali Murad Chandio, District Badin, labeled as haemophilia.

**Patients & Methods:**

Our team visited the village & developed the pedigree of the whole extended family, up to seven generations. Performa was filled by incorporating patients, family history of bleeding, signs & symptoms, and bleeding from any site. From them 144 individuals were screened with CBC, bleeding time, platelet aggregation studies & RiCoF. While for PT, APTT, VWF assay and Factor VIII assay, samples were kept frozen at -70 degrees C until tested.

**Results:**

The family tree of the seven generations comprises of 533 individuals, 63 subjects died over a period of 20 years and 470 were alive. Out of all those 144 subjects were selected on the basis of the bleeding history. Among them 98(68.1%) were diagnosed to have a bleeding disorder; 44.9% patients were male and 55.1% patients were female. Median age of all the patients was 20.81, range (4 months- 80 yrs). The results of bleeding have shown that majority had gum bleeding, epistaxis and menorrhagia. Most common bleeding disorder was Von Willebrand disease and Platelet functional disorders.

**Conclusion:**

Consanguineous marriages keep all the beneficial and adversely affecting recessive genes within the family; in homozygous states. These genes express themselves and result in life threatening diseases. Awareness, education & genetic counseling will be needed to prevent the spread of such common occurrence of these bleeding disorders in the community.

## Introduction

A consanguineous marriage is usually defined as a marriage between people who are second cousins or closer [1]. It is estimated that globally at least 20% of the human population live in communities with a preference for consanguineous marriage and at least 8.5% of children have consanguineous parents [2]. Consanguinity is common in several populations of the world. However, consanguinity rates vary from country to country. In Pakistan close consanguineous unions continue to be extremely common as in South West Asia. The recent "Pakistan Demographic and Health Survey" (DHS) has shown that two-thirds of marriages in Pakistan are consanguineous [3]. As a result of these marriages, the rare autosomal recessive disorders run in close families and tribes. In certain instances many kindred within an extended family or a tribe are commonly seen with phenotypic expression of disease. The subsequent inbreeding, however, leads to increased homozygosity which, in turn, leads to an increased risk of premature morbidity and mortality among the offspring [4-6]. Thus due to autosomal recessive inheritance the rare congenital coagulation bleeding disorders, congenital platelet functional disorders and type III Von Willebrand Disease (VWD) become common. Here we describe a family, (Chandio family, living in village Ali Murad Chandio District Badin) reported by lady health workers, that most of the villagers of both sexes, were suffering from some bleeding disorder most probably haemophilia. This is the first local study of its type in this area, carried out at National Institute of Blood Disease and Bone Marrow Transplantation (NIBD & BMT), Karachi. The information presented in this study describes the risks associated with consanguinity and provides base line data on inheritance of rare bleeding disorder which may help us in formulating a campaign for the awareness of the public in general and the health sector in particular.

## Patients & methods

The study was approved by institutional ethics committee and was done in accordance with declaration of Helsinki [7]. Informed consent was obtained from all adult subjects, parents or legal guardians. It was a cross sectional study. Our team visited the village & developed the pedigree of the whole extended family, up to seven generations. Pre-designed performa was filled by incorporating patient's demographic data, present & past history of bleeding tendency especially at the time of circumcision in male, excessive bleeding from the umbilical stump and history of bleeding in family members. Symptoms and signs were recorded that included petechiae, purpura, echymoses, epistaxis and gum bleed. A detailed history of drug intake was also taken like aspirin, heparin, vancomycin, quinine etc. Individuals on medications affecting platelet functions were excluded from the study. In case of female, maternal and obstetrical history was taken with the help of lady health workers. A group of twenty villagers were called to our centre on alternate days for 03 weeks. 144 individuals visited the center, their previously filled case report form (CRF) forms were reviewed, thorough physical examination was carried out and samples for lab investigations were taken. For coagulation assays, venous blood samples were collected in tubes containing 0.109 M (3.2%) trisodium citrate in a ratio of 9 parts blood to 1 part anticoagulant and then centrifuged without delay at 1200 G for 15 minutes. Complete blood counts (CBC), bleeding time (BT), Prothrombin Time (PT), Activated partial thromboplastin Time (APTT), fibrinogen assay and platelet aggregation studies were carried out on the same day. While for Von Willebrand factor Antigen (VWF: Ag), Ristocetin co-factor assay (RiCoF), Factor VIII & IX assays, samples were collected and kept frozen at -70°C until tested. Blood counts were done on XE-2100 hematology analyzer (Sysmex, Kobe, Japan). Coagulation profile was done on CA-1500 (Sysmex, Kobe, Japan), using appropriate quality control materials and standard reagents (Dade Behring, Germany). PT (normal range: 9.1 - 11.9 sec) and APTT (normal range: 19.98 - 30.93 sec) were performed by employing standard technique while factor assays (normal range: 50-150%) were done by one-stage coagulometric method. Bleeding time (BT) was performed by Ivy's modified template method [8]. Fibrinogen levels were determined by Clauss method (normal range: 2-4 g/l). VWF:Ag was analyzed using Diagnostica Stago reagent, calibrated on Sysmex CA-1500; test was based on immunoturbidimetric method (normal range: 50-150%) while RiCoF (normal range: > 57.0%) was carried out using stabilized platelets agglutinated in the presence of VWF and the antibiotic ristocetin (Agg RAM Helena Laboratories, UK). VWD Classified on the basis of criteria developed by the VWF Subcommittee of the International Society of Thrombosis and Haemostasis (ISTH), first published in 1994 and revised in 2006 [9,10]. The RiCoF to VWF: Ag ratio of < 0.7 was used in differentiating type 1 from type 2 VWD [11]. Type 3 VWD is characterized by undetectable VWF protein and activity, and FVIII levels usually are very low [1-9 IU/dl) [12,13].

Platelet aggregation studies were also done on Helena Agg RAM using ristocetin, adenosine diphosphate (ADP), epinephrine and collagen. Platelet rich plasma (PRP) was used with a count adjusted to 200-250 × 10^9^/L.

Statistical packages for social science (SPSS-13) were used to analyze the data. Frequency and percentage were computed for categorical variables and mean and standard deviation were estimated for quantitative variables.

## Results

**Figure 1:**
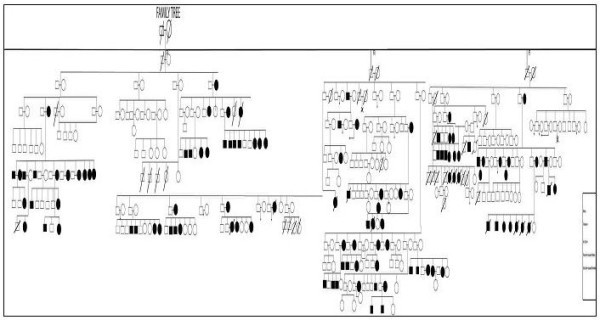
**Pedigree of a family with bleeding**.

Figure 1 shows family tree of the Chandio tribe living in the Ali Murad Chandio village comprising of seven generations. The eldest of their family had 3 sons and 3 daughters who were married to their first and second cousins. The total family tree of the seven generations was comprised of 533 individuals. In such a large family, 63 individuals died over a period of 20 years and 470 were alive. Out of all alive individuals, 144 were selected on the basis of the personal and family history of bleeding in any one of their closely related (mother, father, brother, sister, son, daughter) family members. Out of 144 individuals, 98(68.1%) were diagnosed to have a bleeding disorder on the basis of tests performed; 44 (44.89%) patients were male and 54 (55.10%) were female. Overall median age was 20.81 yrs in both genders, (range 04 months to 80 yrs). In males, median age was 12.4 yrs (range 07 months to 80 yrs); in females, it was 23.34 (range 04 months to 60 yrs). Out of 63 dead family members, cause of death in 16 (25.39%) were excessive bleeding (as per history from close relatives). Among these, 13 were females and 3 were males. Out of 13 females 02 died due to menorrhagia, 03 due to postpartum haemorrhage and 08 at birth. Among males 02 died due to exaggerated bleeding at the time of circumcision and 01 due to fits. Forty seven (74.6%) individuals died due to some other reason or the cause of death was unknown.

**Figure 2:**
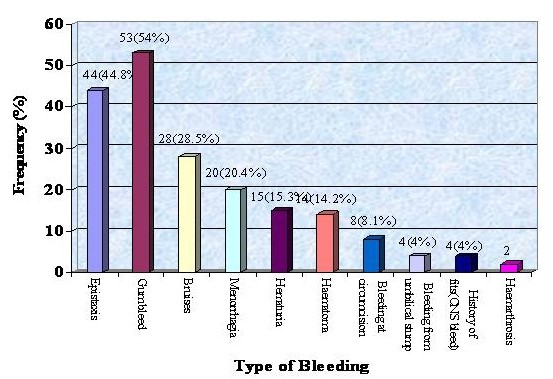
**Type of Bleeding in the Family**.

**Figure 3:**
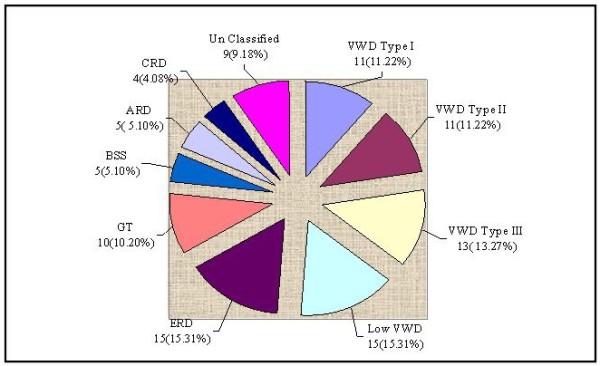
**SPECTRUM OF BLEEDING DISORDERS (n = 98)**. VWD=Von Willebrand disease; ERD = Epinephrine Receptor Defect; GT = Glanzmann's Thrombasthenia; BSS = Bernard Soulier Syndrome; ARD = ADP Receptor Defect; CRD = Collagen Receptor Defect.

Gum bleeding, epistaxis, bruises and menorrhagia were the common bleeding manifestations among the patients of bleeding disorders in the tribe as shown in figure 2. All 144 patients tested showed normal PT 9.4 ± 2.1 sec, no patient had fibrinogen deficiency, nor factor VIII (haemophilia A) or factor IX (haemophilia B) deficiency. Von Willebrand disease was the commonest bleeding disorder, observed in 50 (51.02%) cases followed by 48 (48.98%) cases of platelet functional disorders. In the later group epinephrine receptor defect was noted in 15 (15.31%) cases, Glanzmann's Thrombasthenia (GT) in 10 (10.2%) cases. Cases of Bernard Soulier Syndrome (BSS), adenosine diphosphate (ADP) receptor defect and collagen receptor defect were also found, whereas, in 9 (9.18%) cases, platelet functional disorder could not be classified (figure 3 and table 1).

**Table 1:** Sub Classification of Platelet Aggregation Defects

	ADP	Epinephrine	Collagen	Ristocetin
Epinephrin Receptor Defect,	N	↓	N	N

Glanzman's Thrombasthenia,	↓	↓	↓	N or ↓

Bernard Soulier Syndrome	N	N	N	↓ or Abs

ADP Receptor Defect,	↓	N	N	N

Collagen Receptor Defect.	N	N	↓ or Abs	N

Unclassified	↓ or Abs	↓ or Abs	N	N

ADP- adenosine diphosphate; N - Normal; ↓ - Decrease; Abs - Absent

All patients had positive family history of bleeding tendencies. Among the diagnosed cases of VWD Classified on the basis of criteria developed by the VWF Subcommittee of the ISTH, in 1994 and then in 2006 [8,9]. Table 2 shows the results of VWD patients, VWF:Ag in type-I patients was 33.4% ± 7.1, in type-II, 77.1% ± 18.45 and type III it was 4.59% ± 3.35 (table 2). Similarly average factor VIII concentration in type-I, type-II and type-III VWD were 47.09% ± 13.34, 77.07% ± 19.51 and 9.47% ± 2.95 respectively. Average RiCoF concentration in type-I VWD was 118.76% ± 16.84, type II 27.20% ± 10.58 and 7.98% ± 5.18 in type-III.

**Table 2:** Laboratory Parameters in cases of VWD

Parameter	VWD Type I n = 11	VWD Type II n = 11	VWD Type III n = 13	Low VWF n = 15
Hemoglobin(g/dl)	11.77 ± 2.95	11.11 ± 1.74	8.85 ± 3.06	12.03 ± 2.42
MCV(fl)	64.73 ± 23.61	66.55 ± 8.69	65.46 ± 8.9	66.27 ± 28.78
MCH(pg)	20.45 ± 8.07	25.18 ± 15.61	18.77 ± 5.13	21.13 ± 9.71
Platelet Count(10^9^/L)	297.09 ± 137.87	323.36 ± 79.95	342.31 ± 97.45	254.93 ± 83.72
PT (Sec)	10.41 ± 0.35	10.41 ± 0.69	10.44 ± .56	10.45 ± 0.39
APTT (Sec)	29.65 ± 2.04	28.05 ± 2.66	55.58 ± 4.55	32.31 ± 11.74
Fibrinogen Level (g/dl)	2.89 ± 0.52	3.07 ± 0.43	2.62 ± 0.51	3.00 ± 0.41
VWF: Ag (%)	33.41 ± 7.11	77.10 ± 18.45	4.59 ± 3.35	37.92 ± 4.99
RiCoF (%)	118.76 ± 16.84	27.20 ± 10.58	7.98 ± 5.18	116.03 ± 60.59
Factor VIII (%)	47.09 ± 13.34	77.07 ± 19.51	9.47 ± 2.95	91.71 ± 30.74
BT (Min)	2.73 ± 2.4	2.00 ± 1.09	8.80 ± 1.92	3.00 ± 1.85
RiCoF/VWF:Ag Ratio	2.07 ± 1.02	0.35 ± 0.14	0.28 ± 0.69	3.13 ± 1.91

## Discussion

Pakistan reports a very high proportion of consanguineous marriages similar to that from other Arab countries such as Morocco [14], United Arab Emirates [15], Egypt [16], Lebanon [17], Tunisia [18] Saudi Arabia [19] and non Arab country like Iran [20]. The proportion is significantly higher than in South & North American, European, South African, and Eastern Asian and Oceanic countries [1,2]. Our findings confirm that consanguineous marriages are common among most subgroups in both urban & rural areas in Pakistan and are preferred across all ethnic and religious groups to a varying degree, and that parents continue to be the prime decision-makers for marriages of both sons and daughters. The major reasons for a preference for consanguineous marriages are socio-cultural rather than any perceived economic benefit. The studies by Hussain R et al on consanguineous marriages in Pakistan have shown frequency of 58.7% in the Karachi survey and 62.7% in the DHS. 83.6% of consanguineous marriages in the Karachi survey and 80.4% in the DHS were between first cousins. The mean coefficient of inbreeding in the children of the present generation was 0.0316 in the Karachi study and 0.0331 in the DHS. Actual levels are probably much higher than indicated in these two studies [3,5]. Similarly the study showed odds ratios of perinatal mortality, twice as high among first cousins union or unions with consanguineous parents, implying higher prenatal and/or postnatal losses in couples related as first cousins. The data suggests that premarital genetic counseling and dissemination of information at mass level is needed to increase public awareness about genetic risks associated with consanguineous marriages [3,4,21].

This study shows that autosomal recessive disorders are strongly associated with consanguineous marriages; the consanguinity rate among families with these diseases is found to be significantly different from the consanguinity rate among the general population where the prevalence varies between 1 in 500 000 and 1 in 2 million for the homozygous forms [6]. The effect is particularly marked for rare disorders, because a carrier is unlikely to find a partner who carries the same disorder unless they are related. This means that autosomal recessive genes are hidden within the family for generations and only come to the surface (expressed phenotypically in children) after new consanguineous marriages within the family. In Iran, where the custom of marriages among first cousins is common, recessively inherited coagulation disorders are 3 to 5 times more frequent than in Western countries [22]. Similarly from our neighboring country India reports have also shown a high prevalence rate of rare bleeding disorders [23]. Therefore, in communities where consanguineous marriages are common, there is an increased risk prevalence of many rare bleeding disorders [24].

Peyvandi et al, in a paper on rare coagulation deficiencies, has estimated that in countries where consanguineous marriages are frequent, such as muslim countries and southern India, recessively inherited coagulation deficiencies are so frequent that they can surpass the prevalence of disorders like haemophilia B, representing an important clinical and social problem [6]. The study done by Ahmed et al has shown high frequency of platelet functional disorders (27.77%) as compared to factor IX and other rare coagulation disorder cases in a population of 1576 congenital bleeding disorder patients [25]. Our study of Chandio tribe has shown high prevalence of VWD (51.02%) and platelet functional disorders (48.98%) and no case of haemophilia A or B was detected. VWD is an autosomally inherited disorder resulting from a quantitative or a qualitative defect of Von Willebrand factor (VWF) affecting both genders [9]. Worldwide VWD prevalence is generally 1% of the normal population with higher frequency of type 1 but studies from East has shown higher frequency of type 2 and type 3 as compared to type 1 [25,26]. Menorrhagia is usually the commonest bleeding manifestation in affected female members of the kindred showing as a valuable predictor of a bleeding disorder in women. The frequency of VWD in women with menorrhagia ranges from 5% to 20% in various studies [26-28]. Other common manifestations include mucocutaneous bleed, gum bleed and epistaxis. In severe type 3 VWD patient's presenting features mimic haemophilia i.e., haematoma, haematuria and excessive bleeding at the time of circumcision. We also had 15 patients with low VWF levels (37.92 ± 4.99) below the normal reference established with different blood groups [29].

Inherited platelet functional disorders constitute a large group of rare diseases, involving a wide range of genetic defects that can lead to bleeding symptoms of varying severity [30]. These disorders are relatively more common in communities where consanguineous marriages are more frequent like in the Middle East, India and other developing countries [24,30,31]. This group includes; Glanzmann's thrombasthenia, defects of ADP receptor, epinephrine receptor defect, collagen receptor defect and unclassified [31]. In our study the majority of platelet dysfunction disorders are diagnosed as epinephrine receptor defect (15 cases), Glanzmann's thrombasthenia (10 cases), Bernard-Soulier syndrome (5 cases) and the remaining include collagen receptor defect, ADP receptor defect and unclassified type. GT is a rare disorder caused by a defect in the glycoprotein IIb/IIIa complex receptors. Its incidence has increased in geographic regions where consanguinity is common, like India and Iran [30,32]. BSS, like GT, is characterized by an autosomal recessive pattern of inheritance. It is caused by the deficiency of one of the proteins of the GpIb/IX/V complex, usually GpIb. Our findings show that BSS is very rare as compare to other platelet functional disorders. Recurrent epistaxis has been the main symptom (61.4%) in patients of platelet dysfunction disorders. Among the other common symptoms include excessive bruising, gum bleeding, and prolonged menses. However, in 46 patients we were unable to make diagnosis may be because screening tests for other coagulation factor deficiencies that cause a bleeding disorder, such as hypoprothrombinemia, deficiencies of factor V, combined factor V and factor VIII, factor VII, factor X, and factor XIII, inherited as autosomal recessive traits, were not performed in this study. This necessitates further studies in communities where consanguineous marriages are common and development of expertise with avaibility of specialized tests to classify these disorders.

Thus literature search shows consanguineous marriage is an important part of the cultural and social life of Pakistan. However, the rate of consanguinity seems to be decreasing in frequency among the younger generation particularly among urban educated people [3-5]. The approaches recommended by the WHO to minimize the negative effects of consanguinity on child health should be followed; for example, the identification of families with a high risk of a genetic disease and the provision of prospective genetic counseling. Families with segregating autosomal recessive conditions are usually advised to limit further intermarriages among high at-risk carriers in the family, if carrier testing is not available.

## Conclusion

Consanguineous marriages keep all the beneficial and adversely affecting recessive genes within the family; in homozygous states. These genes express themselves and result in life threatening diseases. Awareness, education & genetic counseling will be needed to prevent the spread of such common occurrence of these rare disorders in the community.

## Competing interests

The authors declare that they have no competing interests.

## Authors' contributions

MB participated in conceiving the study, clinical examination, CRF forms, coordination, manuscript writing and all its editing work. ZP, ZQ, MR visited the village & developed the pedigree of the whole extended family & filled initial CFR forms. AN carried out the laboratory work, AK participated in data entering, developed pedigree and statistical analysis. SA, TF in the sequence alignment, MN helped in editing the manuscript, SAR participated in the design of the study and statistical analysis. TS conceived the study, participated in its design, coordination with NNHF and supervision throughout the study. All authors read and approved the final manuscript.
